# Association of vitamin and/or nutritional supplements with fall among patients with diabetes: A prospective study based on ACCORD and UK Biobank

**DOI:** 10.3389/fnut.2022.1082282

**Published:** 2023-01-13

**Authors:** Lingfang He, Tianqi Ma, Guogang Zhang, Xunjie Cheng, Yongping Bai

**Affiliations:** ^1^Department of Geriatric Medicine, Center of Coronary Circulation, Xiangya Hospital, Central South University, Changsha, China; ^2^National Clinical Research Center for Geriatric Disorders, Xiangya Hospital, Central South University, Changsha, China; ^3^Department of Cardiovascular Medicine, Xiangya Hospital, Central South University, Changsha, China; ^4^Department of Cardiovascular Medicine, The Third Xiangya Hospital, Central South University, Changsha, China

**Keywords:** ACCORD, diabetes, fall, nutritional supplements, UK Biobank

## Abstract

**Aims:**

To assess the associations of vitamin and/or nutritional supplements (VNS) with falls among patients with diabetes.

**Methods:**

9,141 and 21,489 middle-aged participants with diabetes from Action to Control Cardiovascular Risk in Diabetes (ACCORD) trial and UK Biobank were included. Use of VNS was collected at baseline, and fall events were recorded using annual questionnaires in ACCORD and electric records in UK Biobank during follow-up. The associations of VNS use with fall risk were analyzed using logistic regression models in ACCORD and Fine-Gray sub-distribution hazard models in UK Biobank. The role of specific supplements was also estimated in UK Biobank, adjusting for confounding factors and multiple comparisons.

**Results:**

45.9% (4,193/9,141, 5.5 median follow-up years) patients in ACCORD and 10.5% (2,251/21,489, 11.9 median follow-up years) in UK Biobank experienced fall and in-patient events during follow-up, respectively. In ACCORD, VNS using was associated with an increased risk of fall (full-adjusted odds ratio [OR]: 1.26, *P* < 0.05). In UK Biobank, despite no significant association between VNS overall and in-patient fall, vitamin B, calcium, and iron using increased the risk of falls significantly (full-adjusted hazard ratio range: 1.31–1.37, *P* < 0.05).

**Conclusions:**

Use of specific VNS increased the risk of fall among patients with diabetes. The non-indicative use of nutritional supplements for patients with diabetes might be inadvisable.

## 1. Introduction

Fall is a primary burden to older people (1). According to the Global Burden of Diseases Study 2019, falls caused over 0.6 million deaths of all ages globally and were recognized as one of the top-ten-ranking causes of disability-adjusted life-years for older people aged ≥75 years old (2). And fall is tightly associated with poor health outcomes and adverse social effects (3). Nearly 70% of community-dwelling, older adults suffered from physical injuries from a fall, and over one-third reported impaired function after falls (4, 5). Additionally, the worldwide prevalence of diabetes was estimated to be 9.3% in 2019 (6). The prevalent chronic disease increases the risk of falling epidemiologically. According to a meta-analysis by Yang et al. (7), older patients with diabetes had a profoundly increased incidence of falls than diabetes-free people, and a similar tendency was observed among young patients with well-treated type 2 diabetes from the Maastricht Study (8).

The supplementation of vitamin D and calcium has been suggested in multifactorial strategies to prevent injurious falls (9). The preventive role of vitamin supplements, especially vitamin D, in falls has been studied in plenty of randomized, controlled trials (RCTs); however, the conclusions were conflicted. Although in a meta-analysis based on 5 RCTs, Heike et al. (10) found that vitamin D reduced the risk of fall among older people, independent of calcium supplementation, Mark et al. (11) suggested that vitamin D, when were applied as mono-supplement, exhibited no significant effect on falls, hip fractures, and total fractures, irrespective of the doses used. Another recent meta-analysis involving 47 RCTs showed that vitamin D supplementation prevented falls significantly when applied in combination with calcium (12). However, the result that high-dose vitamin D supplementation was associated with a higher risk of falls and fractures was observed (13, 14). Additionally, no other types of vitamin/mineral supplements have been reported to have significant effects on physical function and the risk of fall (15–18). Up till now, most studies about the role of vitamin supplements in falls are RCTs that concentrated on the potential effects of vitamin and/or nutrition treatment in specific types and doses. The settings of these intervention studies are different from the “real world” in which the use of multiple or multi-vitamin/mineral products are common (19). Although routine vitamin supplement is not recommended by current guidelines for patients with diabetes (20, 21), we noticed that nearly 33% of participants in the Action to Control Cardiovascular Risk in Diabetes (ACCORD) trial used vitamin and/or nutritional supplements (VNS) at baseline, and the proportion was 47.0% among participants with diabetes in UK Biobank. These data provide an opportunity to evaluate the potential association between VNS and the risk of fall in patients with diabetes, which has not been addressed in any clinical trials.

Therefore, we performed a *post-hoc* analysis utilizing data from ACCORD trial to assess the association between VNS and fall incidence among patients with type 2 diabetes, verified their associations and specified the role of individual supplements in UK Biobank.

## 2. Materials and methods

### 2.1. Study design and population

Both ACCORD and UK Biobank have been described previously (22, 23). ACCORD trial is a multicenter RCT with a double factorial design that enrolled 10,251 participants aged 40–79 years with type 2 diabetes at a high risk for cardiovascular diseases (CVD) events from 77 clinical sites in the U.S. and Canada. Among 9,620 participants of ACCORD, 9,141 participants with available information in VNS, fall records, and covariates were enrolled in our primary analyses. UK Biobank is a large population-based, prospective cohort study, recruiting over 0.5 million participants aged between 40 and 69 years old from 2006 to 2010 across the U.K. We involved patients with diabetes at baseline (*n* = 27,736) and excluded those with hospital inpatient history of fall (*n* = 810) or missing information in the exposure, outcome, or covariates used (*n* = 5,437), leaving 21,489 participants involved in the second-step analyses. In UK Biobank, the status of diabetes at baseline was derived in aggregation of self-reported medical history, insulin use, and inpatient diagnoses recorded by International Classification of Diseases, 9th revision [ICD-9] and ICD-10 (details in Supplementary Table S1). Analyses based on ACCORD has been approved by the National Heart, Lung, and Blood Institute and the institutional review board of Xiangya Hospital, Central South University. UK Biobank has been approved by the National Health Service (NHS) National Research Ethics Service (approval letter dated 17th June 2011, Ref 11/NW/0382), and this study has been conducted using the UK Biobank resource under the project number 76118. Written informed consents have been provided by the participants in both databases.

### 2.2. Ascertainment of exposures and outcomes

In ACCORD, information on VNS use and fall outcomes were collected from annual questionnaires since 2001, in which participants were asked whether they used VNS or not and the occurrences of any falls in the previous 12 months. In the questionnaires, “Vitamins and/or nutritional supplements” was provided as an option in “Miscellaneous Non-prescribed Therapies,” and fall was defined as “fallen or landed on the floor or ground, or fallen and hit an object like a table or stair.” Participants were classified according to whether they received VNS or not (non-VNS) at baseline. And the occurrences of any falls reported during follow-up were recorded as the outcome.

In UK Biobank, information in VNS use was derived from touchscreen questionnaires finished at recruitment. For vitamin, mineral, and other supplements, participants were asked, “Do you regularly take any of the following? (You can select more than one answer),” with specific supplements including vitamin A, vitamin B, vitamin C, vitamin D, vitamin E, folate (Vit B9), fish oil, glucosamine, calcium, zinc, iron, selenium, and multivitamins ± minerals listed as options. Participants who selected ≥1 supplements were defined with VNS use. Regular use of specific supplements was also recorded according to corresponding options selected. The first occurrence and date of inpatient fall was derived from electric health-related records coded by ICD-9 (E880-E888) and ICD-10 (W00-W19).

### 2.3. Ascertainment of covariates

In ACCORD and UK Biobank, baseline information in demographic factors, lifestyles, and medical history was collected from standardized questionnaires, and anthropometric and biochemical parameters were also measured (details in Supplementary Table S2). In ACCORD, blood pressure, Michigan Neuropathy Screening Instrument (MNSI) score, and Health Utilities Index Mark3 (HUI3) score (an aggregate score of vision, hearing, speech, ambulation, dexterity, emotion, cognition, and pain) were measured by trained technicians. And type 2 diabetes was defined according to the 1997 American Diabetes Association criteria (22). Townsend Deprivation Index was calculated in UK Biobank and reflected the levels of deprivation (24). Body mass index (BMI, kg/m^2^) was calculated as the body weight (kg) divided by the square of height (m^2^). Diet was assessed by a healthy diet score (1 [more advisable]; 0 [less advisable]) adopted from the American Heart Association Guidelines (Supplementary Table S2) (25). For the assessment of frailty status, we used five frailty phenotype items defined by Fried et al. (26, 27): (1) Weight loss; (2) Exhaustion; (3) Physical activity; (4) Walking speed; and (5) Grip strength. The history of other cardiometabolic diseases, osteoporosis, and diabetic complications related to falls at baseline was also ascertained according to self-reported medical history and electrical health records (seen in Supplementary Table S1). In touchscreen questionnaires completed at recruitment, participants were asked, “In the last year have you had any falls?,” and people who answered “Only one fall” or “More than one fall” were thought to have recent fall history.

### 2.4. Statistical analysis

For both ACCORD and UK Biobank, we classified participants according to the use of VNS at baseline, and described the distribution of population characteristics respectively. Continuous variables with normal and abnormal distribution were expressed as mean (standard deviation [SD]) and median (interquartile range [IQR]), respectively, and categorical variables were expressed as number (percentage, %). Unpaired Student *t*-tests, Kruskal tests, and Chi-square tests were applied conditionally to compare the features of participants between the two groups.

In ACCORD trial, the association between VNS and fall outcome was assessed by logistic regression models, since the affirmatory timepoints of outcome were not available. 3 settings of models were fitted: model 1 was adjusted for age, sex, and race, model 2 was additionally adjusted for education, smoking status, and alcohol consumption, and model 3 was further adjusted for BMI, systolic, and diastolic blood pressure, MNSI score, HUI3 score, diabetes duration, level of hemoglobin A1c (HbA1c), history of foot ulcer requiring antibiotics, CVD, heart failure risk, and randomization arms.

Then we verified the association between VNS use and in-patient falls among patients with diabetes in UK Biobank, utilizing Fine-Gray sub-distribution hazard models, which accounted for the competing risk of death. The role of specific supplements including vitamins, mineral, and dietary supplements in the occurrences of in-patient fall was also studied in competing risk models, respectively. The models were fitted with 3 settings: model 1 (adjusted for age, sex, and race); model 2 (additionally adjusted for Townsend Deprivation Index, smoking status, alcohol consumption, diet, sleep duration, and five frailty phenotypes); model 3 (further adjusted for BMI, level of HbA1c, history of other cardiometabolic diseases, osteoporosis, and diabetic complications, and recent falls). To offset the imbalance in distribution of population characteristics in both databases, we calculated propensity scores for the use of VNS using logistic regression, matched the two groups using the nearest neighbor matching method (ratio 1:1), and reperformed the analyses in ACCORD and UK Biobank. In addition, we performed several sensitivity analyses to test the robustness of our results. Firstly, to reduce the potential effects of reverse causality, we excluded participants with self-reported ≥2 falls in the previous year at baseline, since they were at a higher risk of fall constitutionally. Secondly, we substituted the five frailty items with an aggregated variable frailty status. Patients who met no criteria were defined as non-frail, 1 or 2 criteria as pre-frail, and ≥3 criteria as frail. Thirdly, missing information in covariates involved was imputed with multiple imputation by chained equations. Fourthly, we excluded patients with diabetic complications at recruitment. Fifthly, we excluded patients who experienced inpatient fall during the first year of follow-up to account for reverse causality.

All analyses were conducted using R software, version 4.1.0. All *P*-values in our analyses were two-sided, and Benjamini and Hochberg (BH) method was used in analyses based on UK Biobank to account for multiple comparison. It was considered statistically significant when *P*-values < 0.05.

## 3. Results

### 3.1. ACCORD

Of 9,141 enrolled patients, the mean (SD) age was 62.66 (6.60) years and 5,615 (61.4%) were male. Compared with those who did not receive VNS, participants who used VNS were more likely to be female, older, white race, and highly educated; more likely to have higher BMI, lower HbA1c and blood pressure levels (Table 1). A propensity matching was performed and 5,984 participants were identified, with 2,992 in each group (Supplementary Table S3).

**Table 1:** Baseline characteristics of participants in ACCORD and UK Biobank.

**Variable**	**Total sample**	**Non-VNS**	**VNS**	***P***
**ACCORD**				
Participants	9,141	6,053 (66.2)	3,088 (33.8)	–
Male, No. (%)	5,615 (61.4)	3,826 (63.2)	1,789 (57.9)	<0.001
Age, mean (SD), year	62.66 (6.60)	62.40 (6.64)	63.17 (6.51)	<0.001
White, No. (%)	5,744 (62.8)	3,524 (58.2)	2,220 (71.9)	<0.001
Education, No. (%)				<0.001
Less than high school graduate	1,296 (14.2)	997 (16.5)	299 (9.7)	
High school grad (or GED)	2,408 (26.3)	1,650 (27.3)	758 (24.5)	
Some college or technical school	3,006 (32.9)	1,907 (31.5)	1,099 (35.6)	
College graduate or more	2,431 (26.6)	1,499 (24.8)	932 (30.2)	
Current smoker, No. (%)	1,082 (11.8)	795 (13.1)	287 (9.3)	<0.001
Alcohol consumption >2 times/week, No. (%)	1,023 (11.2)	693 (11.4)	330 (10.7)	0.272
MNSI score, median [IQR]	2.00 [0.50, 3.00]	2.00 [0.00, 3.00]	2.00 [1.00, 3.50]	<0.001
HUI3 score, median [IQR]	0.79 [0.59, 0.92]	0.79 [0.59, 0.92]	0.79 [0.58, 0.92]	0.981
BMI, mean (SD), kg/m^2^	32.20 (5.39)	32.02 (5.39)	32.54 (5.39)	<0.001
HbA1c, mean (SD), mmol/mol^a^	67.01 (11.41)	67.66 (11.83)	65.74 (10.40)	<0.001
SBP, mean (SD), mmHg	136.17 (17.03)	136.85 (17.02)	134.84 (16.96)	<0.001
DBP, mean (SD), mmHg	74.92 (10.57)	75.39 (10.61)	73.98 (10.43)	<0.001
Duration of diabetes, mean (SD), year	10.68 (7.50)	10.61 (7.55)	10.82 (7.40)	0.213
History of diseases, No. (%)				
CVD	3,134 (34.3)	2,109 (34.8)	1,025 (33.2)	0.122
Heart failure	407 (4.5)	271 (4.5)	136 (4.4)	0.915
Foot ulcer	305 (3.3)	184 (3.0)	121 (3.9)	0.032
Intervention arms, No. (%)				
Standard glycemia/intensive BP	1,074 (11.7)	719 (11.9)	355 (11.5)	0.700
Standard glycemia/standard BP	1,052 (11.5)	674 (11.1)	378 (12.2)	
Intensive glycemia/intensive BP	1,046 (11.4)	689 (11.4)	357 (11.6)	
Intensive glycemia/standard BP	1,081 (11.8)	728 (12.0)	353 (11.4)	
Standard glycemia/lipid fibrate	1,249 (13.7)	830 (13.7)	419 (13.6)	
Standard glycemia/lipid placebo	1,222 (13.4)	795 (13.1)	427 (13.8)	
Intensive glycemia/lipid fibrate	1,200 (13.1)	809 (13.4)	391 (12.7)	
Intensive glycemia/lipid placebo	1,217 (13.3)	809 (13.4)	408 (13.2)	
**UK Biobank**				
Participants	21,489	11,442 (53.2)	10,047 (46.8)	–
Male, No. (%)	13,109 (61.0)	7,426 (64.9)	5,683 (56.6)	<0.001
Age, median [IQR], year	61.7 [55.6, 65.8]	61.1 [54.6, 65.5]	62.2 [56.7, 66.2]	<0.001
White, No. (%)	19,259 (89.6)	10,365 (90.6)	8,894 (88.5)	<0.001
Townsend deprivation index, median [IQR]	−1.4 [−3.3, 1.8]	−1.4 [−3.2, 1.9]	−1.5 [−3.3, 1.6]	<0.001
Current smoker, No. (%)	2,258 (10.5)	1,340 (11.7)	918 (9.1)	<0.001
Alcohol consumption >2 times/week, No. (%)	6,797 (31.6)	3,677 (32.1)	3,120 (31.1)	0.092
Healthy diet, No. (%)	11,883 (55.3)	5,838 (51.0)	6,045 (60.2)	<0.001
Sleep duration, median [IQR], h/day	7.0 [6.0, 8.0]	7.0 [6.0, 8.0]	7.0 [6.0, 8.0]	0.517
Weight loss, No. (%)	5,949 (27.7)	3,129 (27.3)	2,820 (28.1)	0.244
None or light activity, No. (%)	3,633 (16.9)	2,102 (18.4)	1,531 (15.2)	<0.001
Slow pace, No. (%)	4,770 (22.2)	2,514 (22.0)	2,256 (22.5)	0.405
Low grip score, No. (%)	7,658 (35.6)	3,966 (34.7)	3,692 (36.7)	<0.001
Exhaustion, No. (%)	4,328 (20.1)	2,254 (19.7)	2,074 (20.6)	0.088
BMI, median [IQR], kg/m^2^	30.4 [27.1, 34.4]	30.6 [27.4, 34.6]	30.1 [26.9, 34.2]	<0.001
HbA1c, median [IQR], mmol/mol	49.6 [42.5, 58.7]	50.0 [42.9, 59.6]	49.0 [42.0, 57.7]	<0.001
Fall history, No. (%)	5,358 (24.9)	2,689 (23.5)	2,669 (26.6)	<0.001
History of diseases, No. (%)				
CHD	4,026 (18.7)	2,176 (19.0)	1,850 (18.4)	0.265
Hypertension	15,189 (70.7)	8,148 (71.2)	7,041 (70.1)	0.072
Stroke	1,127 (5.2)	603 (5.3)	524 (5.2)	0.882
Diabetic complications	1,399 (6.5)	726 (6.3)	673 (6.7)	0.308
Osteoporosis	135 (0.6)	55 (0.5)	80 (0.8)	0.005

Data were presented as mean (SD), median [IQR], or *n* (%) for continuous and categorical variables, respectively.

a Only HbA1c values expressed in percentage were available in ACCORD trial, and the values were transformed to mmol/mol in correspondence with the units used in UK Biobank.

ACCORD, action to control cardiovascular risk in diabetes; BMI, body mass index; CHD, coronary heart disease; CVD, cardiovascular disease; DBP, diastolic blood pressure; GED, general educational development; HbA1c, hemoglobin A1c; HUI3, health utilities index mark3; IQR, interquartile range; MNSI, Michigan neuropathy screening instrument; SBP, systolic blood pressure; VNS, vitamin and/or nutritional supplements.

During the median 5.5 years of follow-up, 52.5% (1,620/3,088) and 42.5% (2,573/6,053) patients with and without VNS use occurred at least one fall, respectively. According to the results in total participants (Table 2), using VNS was related to an increased risk of fall among patients with diabetes in model 1 (odds ratio [OR]: 1.32, 95% confidence interval [CI] 1.21–1.45). The association was attenuated but remained significant after multivariable adjustments in model 2 and 3 (full-adjusted OR: 1.26, 95% CI 1.15–1.38). After propensity-matching, VNS using was also significantly associated with an increased risk of fall in patients with diabetes before and after multivariable adjustments (full-adjusted OR: 1.23, 95% CI 1.11–1.37).

**Table 2:** Association between the use of VNS and the risk of fall in ACCORD.

**Participants**	**Exposure group**	**No. of fall events (%)**	**Model 1^a^**		**Model 2^b^**		**Model 3^c^**	
			**OR (95% CI)**	***P***	**OR (95% CI)**	***P***	**OR (95% CI)**	***P***
Total participants	Non-VNS	2,573/6,053 (42.5)	**Reference**		**Reference**		**Reference**	
	VNS	1,620/3,088 (52.5)	**1.32 (1.21, 1.45)**	**<0.001**	**1.31 (1.19, 1.43)**	**<0.001**	**1.26 (1.15, 1.38)**	**<0.001**
Propensity-matched participants	Non-VNS	1,410/2,992 (47.1)	**Reference**		**Reference**		**Reference**	
	VNS	1,558/2,992 (52.1)	**1.22 (1.11, 1.36)**	**<0.001**	**1.22 (1.10, 1.36)**	**<0.001**	**1.23 (1.11, 1.37)**	**<0.001**

a Model 1: adjusted age, sex, and race.

b >Model 2: adjusted for variables in model 1 plus education degree, smoking status lifetime, and alcohol consumption frequency.

c Model 3: adjusted for variables in model 2 plus BMI, SBP, DBP, MNSI score, HUI3 score, duration of diabetes, HbA1c, history of foot ulcer requiring antibiotics, CVD and heart failure at baseline, and randomization Arm.

ACCORD, action to control cardiovascular risk in diabetes; BMI, body mass index; CI, confidence interval; CVD, cardiovascular disease; DBP, diastolic blood pressure; HbA1c, hemoglobin A1c; HUI3, health utilities index mark3; MNSI, Michigan neuropathy screening instrument; OR, odds ratio; SBP, systolic blood pressure; VNS, vitamin and/or nutritional supplements.

Bold values mean statistical significant after adjustment for multiple comparison.

### 3.2. UK Biobank

Characteristics of the participants from UK Biobank are also presented in Table 1. Of 21,489 participants, the median [IQR] age was 61.7 [55.6, 65.8] years and 13,109 (61.0%) were male (Table 1). At baseline, 10,047 (46.8%) patients regularly used VNS, in which 6,071 habitually used vitamins (448 for vitamin A, 1,020 for vitamin B, 1,616 for vitamin C, 738 for vitamin D, 587 for vitamin E, and 617 for folate) and 8,387 habitually used mineral supplements. Compared with non-users, participants who used VNS were more likely to be female, older, non-white and less deprived. They were prone to have healthier lifestyles, fatigue phenotypes, lower BMI, lower HbA1c levels, and fall history. After propensity matching, 18,222 participants were identified, with 9,111 in each group (Supplementary Table S3).

Overall, there were 2,251 participants experienced at least one inpatient fall during 11.9 median follow-up years. Table 3 shows the associations of overall VNS and specific supplements with the risk of inpatient fall in 3 models, respectively. The association between overall VNS and fall appeared not significate (full-adjusted HR: 1.03, 95% CI 0.94–1.12). When further analyzing the role of specific supplements, we observed heterogenicity in their effects on the risk of fall. For instance, after full adjustments, supplementation of total vitamin (HR: 1.10, 95% CI 1.01–1.21), vitamin B (HR: 1.36, 95% CI 1.14–1.61), vitamin D (HR: 1.27, 95% CI 1.03–1.56), folate (HR: 1.23, 95% CI 1.00–1.53), calcium (HR: 1.37, 95% CI 1.17–1.60), iron (HR: 1.33, 95% CI 1.10–1.60), and selenium (HR: 1.37, 95% CI 1.05–1.78) was associated with a higher risk of inpatient fall, respectively (Table 3). After further adjustment for multiple comparisons, the associations with fall risk remained significant for vitamin B (adjusted *P* = 0.004), calcium (adjusted *P* = 0.002), and iron (adjusted *P* = 0.015). No significant effect was observed for other specific supplements including vitamin A, vitamin C, vitamin D, vitamin E, folate, total mineral supplement, fish oil, glucosamine, zine, and selenium (adjusted *P* > 0.05). After propensity matching, the effects of vitamin B (full-adjusted HR: 1.35, adjusted *P* = 0.009) and calcium (full-adjusted HR: 1.36, adjusted *P* = 0.008) remained significant (Supplementary Table S4).

**Table 3:** Associations of overall VNS and specific VNS with the risk of fall in UK Biobank.

**Main variable**	**No. of fall events (%)**		**Model 1^a^**		**Model 2^b^**		**Model 3^c^**	
	**Used**	**Non-used**	**HR (95% CI)**	***P* ^d^**	**HR (95% CI)**	***P* ^d^**	**HR (95% CI)**	***P* ^d^**
Overall VNS	1,092/10,047 (10.87)	1,159/11,442 (10.13)	1.00 (0.92, 1.09)	0.950	1.03 (0.95, 1.12)	0.554	1.03 (0.94, 1.12)	0.623
Vitamin	680/6,071 (11.20)	1,571/15,418 (10.19)	1.10 (1.00, 1.20)	0.073	1.12 (1.02, 1.22)	0.051	1.10 (1.01, 1.21)	0.095
Vitamin A	54/448 (12.05)	2,197/21,041 (10.44)	1.17 (0.90, 1.53)	0.327	1.16 (0.89, 1.52)	0.395	1.13 (0.86, 1.49)	0.518
Vitamin B	139/1,020 (13.63)	2,112/20,469 (10.32)	**1.39 (1.17, 1.65)**	**<0.001**	**1.39 (1.17, 1.65)**	**0.001**	**1.36 (1.14, 1.61)**	**0.004**
Vitamin C	171/1,616 (10.58)	2,080/19,873 (10.47)	1.00 (0.85, 1.16)	0.950	1.04 (0.89, 1.22)	0.632	1.02 (0.87, 1.19)	0.820
Vitamin D	95/738 (12.87)	2,156/20,751 (10.39)	**1.27 (1.03, 1.56)**	**0.006**	1.29 (1.05, 1.58)	0.051	1.27 (1.03, 1.56)	0.069
Vitamin E	69/587 (11.75)	2,182/20,902 (10.44)	1.11 (0.88, 1.41)	0.475	1.17 (0.93, 1.49)	0.300	1.18 (0.93, 1.49)	0.283
Folate	89/617 (14.42)	2,162/20,872 (10.36)	**1.44 (1.16, 1.78)**	**0.003**	1.27 (1.03, 1.57)	0.060	1.23 (1.00, 1.53)	0.116
Mineral supplements	885/8,387 (10.55)	1,366/13,102 (10.43)	0.92 (0.85, 1.00)	0.095	0.96 (0.88, 1.05)	0.438	0.96 (0.88, 1.05)	0.510
Fish oil	630/6,220 (10.13)	1,621/15,269 (10.62)	0.87 (0.79, 0.95)	0.072	0.93 (0.85, 1.02)	0.206	0.93 (0.85, 1.02)	0.244
Glucosamine	309/2,958 (10.45)	1,942/18,531 (10.48)	0.88 (0.78, 0.99)	0.073	0.94 (0.83, 1.06)	0.395	0.96 (0.85, 1.08)	0.613
Calcium	168/1,102 (15.25)	2,083/20,387 (10.22)	**1.45 (1.23, 1.70)**	**<0.001**	**1.42 (1.21, 1.66)**	**<0.001**	**1.37 (1.17, 1.60)**	**0.002**
Iron	118/799 (14.77)	2,133/20,690 (10.31)	**1.59 (1.33, 1.92)**	**<0.001**	**1.41 (1.17, 1.69)**	**0.002**	**1.33 (1.10, 1.60)**	**0.015**
Zine	74/753 (9.83)	2,177/20,736 (10.50)	0.92 (0,73, 1.16)	0.565	0.98 (0.78, 1.23)	0.840	0.97 (0.77, 1.22)	0.820
Selenium	56/412 (13.59)	2,195/21,077 (10.41)	1.24 (0.96, 1.61)	0.150	1.35 (1.03, 1.75)	0.060	1.37 (1.05, 1.78)	0.069

a Model 1: adjusted for age, gender, and race.

b Model 2: adjusted for age, gender, race, Townsend deprivation index, smoking statue, alcohol consumption, diet, sleep duration, activity, weight loss, grip strength score, walk speed, and exhaustion.

c Model 3: adjusted for variables in model 2 plus BMI, HbA1c, history of CHD, hypertension, stroke, osteoporosis, and diabetic complication, and recent fall.

d *P*: *P*-value was adjusted for multiple comparisons using Benjamini&Hochberg method.

BMI, body mass index; CHD, coronary heart disease; CI, confidence interval; HbA1c, hemoglobin A1c; HR, hazard ratio; VNS, vitamin and/or nutritional supplements.

Bold values mean statistical significant after adjustment for multiple comparison.

In sensitivity analyses, similar tendency was observed for the associations of overall VNS and specific supplements with the risk of inpatient falls, after excluding the participants who had a previous fall history, reported diabetic complications, occurred fall events during the first year of follow-up, modifying the assessment of frailty phenotype, or imputing the missing information in covariates (Supplementary Table S5).

## 4. Discussion

In this study, we assessed the associations of VNS use with the risk of fall among elderly patients with diabetes. Based on data from the ACCORD trial, we observed that VNS using was associated with an increased risk of fall events among patients with diabetes, and further tested our findings for specific supplements utilizing UK Biobank. We found that supplementation of vitamin B, iron, and calcium was independently associated with a higher risk of inpatient fall among patients with diabetes.

Existing studies in the effects of nutritional supplementation on falls mainly focused on vitamin D, generating mixed results. For instance, Bolland et al. (11) enrolled 37 RCTs, performed a trial sequential analysis, and found that vitamin D, when were applied as mono-supplement, exhibited no significant effect on falls, hip fractures, and total fractures, and the effects of vitamin D were independent of the doses used, while a recent meta-analysis indicated the protective role of vitamin D in fall prevention, especially when combined with calcium (12). Further analyses suggested that its effects might vary according to the supplementation doses and baseline nutritional status of participants (28, 29). Additionally, several RCTs have been conducted to evaluate the effects of vitamin B12 and folic acid supplementation (18) or vitamin K (16) on falls, and no significant effects on the risk of falls were observed. Although previous studies showed that most nutritional supplementation was associated with an increased muscle strength and alleviated frailty, there has been no studies that systematically evaluate the role of vitamins and dietary supplements on falls. And for patients with diabetes, who are at a higher risk of falls, no specific fall-preventive strategies have been developed yet. Therefore, utilizing data from the ACCORD trial and UK Biobank, we assessed the effects of total VNS, vitamins, and specific supplements on the risk of falls among patients with diabetes, and found the heterogenicity in their implications. Among the vitamins and dietary supplements involved in the baseline questionnaires of UK Biobank, vitamin B, calcium, and iron supplementation were associated with a significantly increased risk of inpatient fall, while the associations of other supplements with fall appeared insignificant, which appeared inconsistent with previous studies examining the associations of VNS with falls.

For fall-related clinical characteristics such as skeletal muscle mass, bone health, balance function, and frailty, the effects of nutritional supplementation observed in existing research also seemed to contradict our results (30). In a prospective study which enrolled 1,643 community-dwelling individuals aged ≥65 years, poor intake of vitamin B6, C, E, and folates was strongly and independently associated with frailty (31), and supplementation of whey protein, vitamin D, and E improved the muscle mass, strength, and life quality in older adults with sarcopenia (32). For elderly people, folate levels predict grip and leg strength (33), and low folate concentration is a risk factor for worse physical performance (34). Also, low levels of vitamin C and iron are also associated with age-related muscle loss and frailty syndrome (35–37). However, in reviewing the published literature, we also found that most studies that observed protective role of nutritional supplements in falls and related mechanisms were conducted among participants with nutritional deficiency. In developed countries such as U.K., U.S., and Canada, nutrient intake can usually be fulfilled by a high-quality diet, and people without increased demands might not benefit additionally *via* an extra nutritional supplementation (38–40). In addition, excessive nutritional supplementation might also have side effects or toxicity. For example, previous RCTs reported that supplementation of vitamin D in high doses daily (4,000 or 4,800 IU vitamin D3), monthly (60,000 IU vitamin D3 or 24,000 IU vitamin D3 plus 300 μg calcifediol), or annually (500,000 IU cholecalciferol) increased the risk of falls (13, 14, 41). High-dose vitamin B supplementation was associated with an increased risk for cataract (42). And in experimental studies, iron overload increased bone reabsorption and caused bone loss in mouse models (43). The evidence mentioned above supports our findings that specific vitamin and nutritional supplements were associated with an increased risk of inpatient fall among patients with diabetes. In addition, the information in the doses, duration, and brands for specific nutritional supplementation was not available in ACCORD and UK Biobank, and whether or not the enrolled participants had specific nutrient deficiencies was unknown in our analyses. Thus, it might be inapposite to compare our findings, which were derived from a “real-world” setting, with those of previous RCTs of specific supplements directly. And based on UK Biobank, we also conducted a series of sensitivity analyses and verified the reliability and robustness of our findings that vitamin B, iron, and calcium were independently associated with a higher risk of inpatient fall among patients with diabetes.

Furthermore, diabetes is a non-negligible risk factor for falls (7), which is attributed to a series of pathophysiological decline (impaired postural control, gait patterns, and cognitive function), diabetic complications (polyneuropathy, dysfunction of visual and vestibular systems), and other co-morbidities such as hypertension, hyperlipemia, and CVDs that might appear along with the progression of diabetes (44). Considering that diabetes-related frailty, muscle strength reduction, retinopathy, peripheral neuropathy, and other pathophysiological mechanisms involved in the diabetes-related higher risk of falls, we excluded patients with fall-related diabetic complications at baseline in sensitivity analysis 4 to avoid the related reverse causality, and the results remained consistent. Although the European Society of Cardiology and the European Association for the Study of Diabetes do not recommend the general use of vitamin or micronutrient supplementation to prevent diabetes or CVDs for patients with diabetes, we observed that nutritional supplementation was common among patients with diabetes in both ACCORD trial (33.8%) and UK Biobank (46.8%). According to a recent research based on the National Health and Nutrition Examination Surveys, ~58% of U.S. adults with diabetes used dietary supplements during 2013–2014 (45). Based on this phenomenon, our results, although did not assess the dose-dependent effects of each supplement, are still important for patients, clinicians, and policymakers. For patients without malnutrition or special demands, the non-indicative use of dietary supplements needs to be carefully evaluated, since its side effects might lead to worse prognosis, despite its undefined clinical benefits. Utilizing the two prospective data with “real-world” settings, this study provides epidemiological evidence to warn the non-indicative use of nutritional supplements for patients with diabetes, considering its potentially higher risk of falls. And further clinical trials and genetically epidemiological research are necessary to prove our findings and provide higher-level evidence.

There were several limitations to our study. Firstly, the use of VNS was self-reported by participants at baseline in two cohorts. The detailed doses, duration, and brands of the supplements used were not available, making it impossible to analyze the potential dose-dependent effects of specific supplements. And during follow-up period, possible alterations in the exposure level might contribute to dilution bias in our analyses. However, according to the published studies, the reproducibility of nutritional supplementation in UK Biobank was stable and reliable (46, 47). Secondly, since the incidence of general fall events (e.g., any falls in the last year) during follow-up was only available for a subset of participants and the corresponding date of self-reported fall events was unavailable in UK Biobank, we defined the fall outcome as inpatient fall event using ICD-10 codes and corresponding date derived from electrical health records by UK Biobank, which might underestimate the incidence of fall events. Thirdly, the two cohorts used in this study, ACCORD and UK Biobank, were based on the developed countries, and most participants were white. Thus, it needs caution to generalize our findings to the population from other developing countries and races. Fourthly, reverse causality might remain in this observational research, although we have attempted to avoid its impacts by conducting several sensitivity analyses. For instance, in sensitivity analysis 1, after exclusion of participants who experienced ≥2 falls in the previous year, consistent results were also observed. Finally, even though we have adjusted for potential confounding factors including fall-related diabetic complications, frailty phenotype, previous fall history, and osteoporosis in the multivariable models, there were still residual confounding factors (e.g., balance measurements, not available in ACCORD and UK Biobank), which might cause deviations in our results.

In summary, our study revealed that specific nutritional supplementation of vitamin B, iron, and calcium were significantly associated with an increased risk of fall among patients with diabetes after adjustments for demographic, clinical, and laboratory factors, and further randomized, controlled trials designed specifically are in demand to validate our conclusion further.

## Data availability statement

The datasets analyzed for this study can be found in the National Heart, Lung, and Blood Institute Biologic Specimen and Data Repository (https://biolincc.nhlbi.nih.gov/studies/accord/ for ACCORD trial) and UK Biobank (https://www.ukbiobank.ac.uk/) but restrictions apply to the availability of these data, which were used under license for the current study, and so are not publicly available. Data are however available from the authors upon reasonable request and with permission of the National Heart, Lung, and Blood Institute Biologic Specimen and Data Repository and UK Biobank.

## Ethics statement

The studies involving human participants were reviewed and approved by the National Heart, Lung, and Blood Institute the Institutional Review Board of Xiangya Hospital, Central South University the National Health Service (NHS) National Research Ethics Service. The patients/participants provided their written informed consent to participate in this study.

## Author contributions

YB and XC are the guarantor of this work, had full access to all original data in the study, take responsibility for the integrity of the data, and the accuracy of the data analysis in the study. XC researched data. LH and TM researched data and wrote the manuscript. GZ, XC, and YB contributed to the discussion and reviewed/edited the manuscript. All authors contributed to the article and approved the submitted version.

## References

[1] Bergen G Stevens MR Burns ER. Falls and fall injuries among adults aged ≥65 years—United States, 2014. MMWR Morb Mortal Wkly Rep. (2016) 65:993–8. 10.15585/mmwr.mm6537a2 27656914

[2] Diseases GBD Injuries C. Global burden of 369 diseases and injuries in 204 countries and territories, 1990–2019: a systematic analysis for the global burden of disease study 2019. Lancet. (2020) 396:1204–22. 10.1016/S0140-6736(20)30925-9 33069326 PMC7567026

[3] Ambrose AF Paul G Hausdorff JM. Risk factors for falls among older adults: a review of the literature. Maturitas. (2013) 75:51–61. 10.1016/j.maturitas.2013.02.009 23523272

[4] Stel VS Smit JH Pluijm SM Lips P. Consequences of falling in older men and women and risk factors for health service use and functional decline. Age Ageing. (2004) 33:58–65. 10.1093/ageing/afh028 14695865

[5] Alamgir H Muazzam S Nasrullah M. Unintentional falls mortality among elderly in the United States: time for action. Injury. (2012) 43:2065–71. 10.1016/j.injury.2011.12.001 22265137

[6] Saeedi P Petersohn I Salpea P Malanda B Karuranga S Unwin N. Global and regional diabetes prevalence estimates for 2019 and projections for 2030 and 2045: results from the international diabetes federation diabetes Atlas, 9(Th) edition. Diabetes Res Clin Pract. (2019) 157:107843. 10.1016/j.diabres.2019.107843 31518657

[7] Yang Y Hu X Zhang Q Zou R. Diabetes mellitus and risk of falls in older adults: a systematic review and meta-analysis. Age Ageing. (2016) 45:761–7. 10.1093/ageing/afw140 27515679

[8] de Waard EAC Koster A Melai T van Geel TA Henry RMA Schram MT. The association between glucose metabolism status, diabetes severity and a history of fractures and recent falls in participants of 50 years and older-the Maastricht study. Osteoporos Int. (2016) 27:3207–16. 10.1007/s00198-016-3645-0 27234668 PMC5059422

[9] Tricco AC Thomas SM Veroniki AA Hamid JS Cogo E Strifler L. Comparisons of interventions for preventing falls in older adults: a systematic review and meta-analysis. JAMA. (2017) 318:1687–99. 10.1001/jama.2017.15006 29114830 PMC5818787

[10] Bischoff-Ferrari HA Dawson-Hughes B Willett WC Staehelin HB Bazemore MG Zee RY. Effect of vitamin D on falls: a meta-analysis. JAMA. (2004) 291:1999–2006. 10.1001/jama.291.16.1999 15113819

[11] Bolland MJ Grey A Avenell A. Effects of vitamin D supplementation on musculoskeletal health: a systematic review, meta-analysis, and trial sequential analysis. Lancet Diabetes Endocrinol. (2018) 6:847–58. 10.1016/S2213-8587(18)30265-1 30293909

[12] Thanapluetiwong S Chewcharat A Takkavatakarn K Praditpornsilpa K Eiam-Ong S Susantitaphong P. Vitamin D supplement on prevention of fall and fracture: a meta-analysis of randomized controlled trials. Medicine. (2020) 99:e21506. 10.1097/MD.0000000000021506 32846760 PMC7447507

[13] Sanders KM Stuart AL Williamson EJ Simpson JA Kotowicz MA Young D. Annual high-dose oral vitamin d and falls and fractures in older women: a randomized controlled trial. JAMA. (2010) 303:1815–22. 10.1001/jama.2010.594 20460620

[14] Bischoff-Ferrari HA Dawson-Hughes B Orav EJ Staehelin HB Meyer OW Theiler R. Monthly high-dose vitamin d treatment for the prevention of functional decline: a randomized clinical trial. JAMA Intern Med. (2016) 176:175–83. 10.1001/jamainternmed.2015.7148 26747333

[15] Dangour AD Albala C Allen E Grundy E Walker DG Aedo C. Effect of a nutrition supplement and physical activity program on pneumonia and walking capacity in Chilean older people: a factorial cluster randomized trial. PLoS Med. (2011) 8:e1001023. 10.1371/journal.pmed.1001023 21526229 PMC3079648

[16] Witham MD Price RJG Band MM Hannah MS Fulton RL Clarke CL. Effect of vitamin K2 on postural sway in older people who fall: a randomized controlled trial. J Am Geriatr Soc. (2019) 67:2102–7. 10.1111/jgs.16024 31211416 PMC6851824

[17] Ng TP Feng L Nyunt MS Feng L Niti M Tan BY. Nutritional, physical, cognitive, and combination interventions and frailty reversal among older adults: a randomized controlled trial. Am J Med. (2015) 128:1225–36.e1. 10.1016/j.amjmed.2015.06.017 26159634

[18] Swart KM Ham AC van Wijngaarden JP Enneman AW van Dijk SC Sohl E. A randomized controlled trial to examine the effect of 2-year vitamin B12 and folic acid supplementation on physical performance, strength, and falling: additional findings from the B-proof study. Calcif Tissue Int. (2016) 98:18–27. 10.1007/s00223-015-0059-5 26412463 PMC4703626

[19] Ford KL Jorgenson DJ Landry EJL Whiting SJ. Vitamin and mineral supplement use in medically complex, community-living, older adults. Appl Physiol Nutr Metab. (2019) 44:450–3. 10.1139/apnm-2018-0515 30628461

[20] American Diabetes Association. 5. Facilitating behavior change and well-being to improve health outcomes: standards of medical care in diabetes-2020. Diabetes Care. (2020) 43(Suppl 1):S48–65. 10.2337/dc20-S005 31862748

[21] Cosentino F Grant PJ Aboyans V Bailey CJ Ceriello A Delgado V. 2019 Esc guidelines on diabetes, pre-diabetes, and cardiovascular diseases developed in collaboration with the Easd. Eur Heart J. (2020) 41:255–323. 10.1093/eurheartj/ehz486 31497854

[22] Group AS Buse JB Bigger JT Byington RP Cooper LS Cushman WC. Action to control cardiovascular risk in diabetes (Accord) trial: design and methods. Am J Cardiol. (2007) 99:21i−33i. 10.1016/j.amjcard.2007.03.003 17599422

[23] Sudlow C Gallacher J Allen N Beral V Burton P Danesh J. UK Biobank: an open access resource for identifying the causes of a wide range of complex diseases of middle and old age. PLoS Med. (2015) 12:e1001779. 10.1371/journal.pmed.1001779 25826379 PMC4380465

[24] Ye J Wen Y Sun X Chu X Li P Cheng B. Socioeconomic deprivation index is associated with psychiatric disorders: an observational and genome-wide gene-by-environment interaction analysis in the UK Biobank cohort. Biol Psychiatry. (2021) 89:888–95. 10.1016/j.biopsych.2020.11.019 33500177

[25] Benjamin EJ Blaha MJ Chiuve SE Cushman M Das SR Deo R. Heart disease and stroke statistics-2017 update: a report from the American heart association. Circulation. (2017) 135:e146–603. 10.1161/CIR.0000000000000491 28122885 PMC5408160

[26] Fried LP Tangen CM Walston J Newman AB Hirsch C Gottdiener J. Frailty in older adults: evidence for a phenotype. J Gerontol A Biol Sci Med Sci. (2001) 56:M146–56. 10.1093/gerona/56.3.M146 11253156

[27] Hanlon P Nicholl BI Jani BD Lee D McQueenie R Mair FS. Frailty and pre-frailty in middle-aged and older adults and its association with multimorbidity and mortality: a prospective analysis of 493,737 UK Biobank participants. Lancet Public Health. (2018) 3:e323–e32. 10.1016/S2468-2667(18)30091-4 29908859 PMC6028743

[28] Ling Y Xu F Xia X Dai D Xiong A Sun R. Vitamin D supplementation reduces the risk of fall in the vitamin D deficient elderly: an updated meta-analysis. Clin Nutr. (2021) 40:5531–7. 10.1016/j.clnu.2021.09.031 34656949

[29] Kong SH Jang HN Kim JH Kim SW Shin CS. Effect of vitamin D supplementation on risk of fractures and falls according to dosage and interval: a meta-analysis. Endocrinol Metab. (2022) 37:344–58. 10.3803/EnM.2021.1374 35504603 PMC9081312

[30] Gana W De Luca A Debacq C Poitau F Poupin P Aidoud A. Analysis of the impact of selected vitamins deficiencies on the risk of disability in older people. Nutrients. (2021) 13:163. 10.3390/nu13093163 34579039 PMC8469089

[31] Balboa-Castillo T Struijk EA Lopez-Garcia E Banegas JR Rodriguez-Artalejo F Guallar-Castillon P. Low vitamin intake is associated with risk of frailty in older adults. Age Ageing. (2018) 47:872–9. 10.1093/ageing/afy105 30052701

[32] Bo Y Liu C Ji Z Yang R An Q Zhang X. A high whey protein, vitamin D and E supplement preserves muscle mass, strength, and quality of life in sarcopenic older adults: a double-blind randomized controlled trial. Clin Nutr. (2019) 38:159–64. 10.1016/j.clnu.2017.12.020 29395372

[33] Wee AK. Serum folate predicts muscle strength: a pilot cross-sectional study of the association between serum vitamin levels and muscle strength and gait measures in patients >65 years old with diabetes mellitus in a primary care setting. Nutr J. (2016) 15:89. 10.1186/s12937-016-0208-3 27756315 PMC5070191

[34] Ao M Inuiya N Ohta J Kurose S Takaoka H Abe Y. Relationship between homocysteine, folate, vitamin B12 and physical performance in the institutionalized elderly. J Nutr Sci Vitaminol. (2019) 65:1–7. 10.3177/jnsv.65.1 30814404

[35] Lewis LN Hayhoe RPG Mulligan AA Luben RN Khaw KT Welch AA. Lower dietary and circulating vitamin c in middle- and older-aged men and women are associated with lower estimated skeletal muscle mass. J Nutr. (2020) 150:2789–98. 10.1093/jn/nxaa221 32851397 PMC7549302

[36] Johnston CS Barkyoumb GM Schumacher SS. Vitamin C supplementation slightly improves physical activity levels and reduces cold incidence in men with marginal vitamin C status: a randomized controlled trial. Nutrients. (2014) 6:2572–83. 10.3390/nu6072572 25010554 PMC4113757

[37] Zawadzki B Mazur G Butrym A. Iron dysregulation and frailty syndrome. J Clin Med. (2021) 10:5596. 10.3390/jcm10235596 34884301 PMC8658196

[38] Rautiainen S Manson JE Lichtenstein AH Sesso HD. Dietary supplements and disease prevention: a global overview. Nat Rev Endocrinol. (2016) 12:407–20. 10.1038/nrendo.2016.54 27150288

[39] Bischoff-Ferrari HA Freystatter G Vellas B Dawson-Hughes B Kressig RW Kanis JA. Effects of vitamin D, omega-3 fatty acids, and a simple home strength exercise program on fall prevention: the do-health randomized clinical trial. Am J Clin Nutr. (2022) 115:1311–21. 10.1093/ajcn/nqac022 35136915

[40] Fingeret M Vollenweider P Marques-Vidal P. No association between vitamin C and E supplementation and grip strength over 5 years: the Colaus study. Eur J Nutr. (2019) 58:609–17. 10.1007/s00394-018-1646-9 29484474

[41] Smith LM Gallagher JC Suiter C. Medium doses of daily vitamin D decrease falls and higher doses of daily vitamin D3 increase falls: a randomized clinical trial. J Steroid Biochem Mol Biol. (2017) 173:317–22. 10.1016/j.jsbmb.2017.03.015 28323044 PMC5595629

[42] Selin JZ Lindblad BE Bottai M Morgenstern R Wolk A. High-dose B-vitamin supplements and risk for age-related cataract: a population-based prospective study of men and women. Br J Nutr. (2017) 118:154–60. 10.1017/S0007114517001994 28820082

[43] Tsay J Yang Z Ross FP Cunningham-Rundles S Lin H Coleman R. Bone loss caused by iron overload in a murine model: importance of oxidative stress. Blood. (2010) 116:2582–9. 10.1182/blood-2009-12-260083 20554970 PMC2953890

[44] Rasmussen NH Dal J. Falls and fractures in diabetes-more than bone fragility. Curr Osteoporos Rep. (2019) 17:147–56. 10.1007/s11914-019-00513-1 30915638

[45] Li J Li X Gathirua-Mwangi W Song Y. Prevalence and trends in dietary supplement use among us adults with diabetes: the national health and nutrition examination surveys, 1999–2014. BMJ Open Diabetes Res Care. (2020) 8:925. 10.1136/bmjdrc-2019-000925 31958304 PMC7039581

[46] Liu X Zhuang P Li Y Wu F Wan X Zhang Y. Association of fish oil supplementation with risk of incident dementia: a prospective study of 215,083 older adults. Clin Nutr. (2022) 41:589–98. 10.1016/j.clnu.2022.01.002 35124466

[47] Carter JL Lewington S Piernas C Bradbury K Key TJ Jebb SA. Reproducibility of dietary intakes of macronutrients, specific food groups, and dietary patterns in 211,050 adults in the UK Biobank study. J Nutr Sci. (2019) 8:e34. 10.1017/jns.2019.31 31723428 PMC6842574

