# Supplementary material for: Association of vitamin and/or nutritional supplements with fall among patients with diabetes: A prospective study based on ACCORD and UK Biobank
Source: Front Nutr. 2023 Jan 13;9:1082282. doi: 10.3389/fnut.2022.1082282 (PMC9880286; doi:10.3389/fnut.2022.1082282)
Supplement: Supplementary file 1 [file Data_Sheet_1.docx]

Supplementary Material

Table S1. Diseases Definitions in UK Biobank

| Diseases | Self-reported information | ICD-9 information | ICD-10 information | OPCS-4 information |
| --- | --- | --- | --- | --- |
| Diabetes | 2443(1), 2976, 6153(3), 6177(3), 20002(1220, 1222, 1223), 20008, 20009 | 41271 (250, 3572, 3620), 41281 | 41270 (E10-E14, G590, G632, H280, H360, M142, N083), 41280, 130714, 130712, 130710, 130708, 130706, 130715, 130713, 130711, 130709, 130707 |  |
| Diabetic complications | 20002(1276, 1468) | 41271 (2504, 2505, 3572, 3620), 41281 | 41270 (G590, G632, H280, H360), 41280 |  |
| Hypertension | 2966, 6150(4), 6153(2), 6177(2), 20002(1065, 1072), 20008, 20009 | 41271 (401-405), 41281 | 41270 (I10-I13, I15, O10), 41280, 131292, 131290, 131288, 131286, 131294, 132180, 131295, 131293, 131291, 131289, 131287, 132181 |  |
| CHD | 6150(1, 2), 3894, 3627, 20004(1070, 1095, 1523), 20002(1074, 1075), 20008, 20009, 20010, 20011 | 41271 (410-414), 41281 | 41270 (I20-I25, Z951, Z955), 41280, 131296, 131298, 131300, 131302, 131304, 131306, 131307, 131305, 131303, 131301, 131299, 131297 | 41272 (K40-K46, K49, K50, K75), 41282 |
| Stroke | 6150(3), 4056, 20002(1081, 1491, 1583, 1086), 20008, 20009, 20010, 20011 | 41271 (3361, 36231, 36232, 430, 431, 4329, 43301, 43311, 43321, 43331, 43381, 43391, 434, 436), 41281 | 41270 (I60, I61, I629, I63, I64, I678, I690, I693, G951, H341, H342, S066), 41280, 131378, 131376, 131374, 131372, 131370, 131368, 131366, 131364, 131362, 131360, 131180, 131379, 131377, 131375, 131373, 131371, 131369, 131367, 131365, 131363, 131361, 131181 | 41272 (A052-A054, L351, L353, L343), 41282 |
| Osteoporosis | --- | --- | 41270 (M80, M81, M82) | --- |

The filed IDs of the self-reported information and medical records in the UK Biobank, which were used to define the prevalence of diabetes, diabetic complications related to falls, and other cardiometabolic diseases in individuals are presented.

CHD: coronary heart disease; ICD: International Classification of Diseases; OPCS: Office of Population Censuses and Surveys Classification of Interventions and Procedures.

Table S2. Covariates definition in ACCORD and UK Biobank

| Covariates | Definition |
| --- | --- |
| ACCORD |  |
| Age | Continuous variable (year). |
| Sex | Binary variable: male and female. |
| Race | Categorical variable: White, Black, Hispanic, and Other. |
| Education | Participant’s highest level of education. Categorical variable: Less than high school graduate, High school grad (or GED), Some college or technical school, College graduate or more. |
| Current smoking status | Binary variable: current cigarette smoker or not. |
| Alcohol consumption frequency | Binary variable: >2 times/week and ≤2 times/week. |
| MNSI score | Michigan Neuropathy Screening Instrument score, measured at baseline. Continuous variable. |
| HUI3 score | Aggregate score of vision, hearing, speech, ambulation, dexterity, emotion, cognition and pain. Continuous variable. |
| BMI | Body weight (kg) divided by the square of height (m^2^). Continuous variable (kg/m^2^). |
| HbA1c | Laboratory measures at baseline. The values expression in % provided in ACCORD were transformed to mmol/mol in accordance to the expression in UK Biobank. Continuous variable (mmol/mol). |
| SBP | Measured at baseline. Continuous variable (mmHg). |
| DBP | Measured at baseline. Continuous variable (mmHg). |
| Duration of diabetes | Year of diabetes diagnosis. Continuous variable (year). |
| History of CVD | Binary variable: yes and no. |
| History of heart failure | Binary variable: yes and no. |
| History of fool ulcer | Foot ulcer requiring antibiotics. Binary variable: yes and no. |
| UK Biobank |  |
| Age | Continuous variable (year). |
| Sex | Binary variable: male and female. |
| Race | Binary variable: white and non-white. |
| Townsend Deprivation Index | Continuous variable, an integrated indicator of socioeconomic status calculated from participants’ home postcodes. |
| Current smoking status | Binary variable: current smoking and non-current smoking. |
| Alcohol consumption frequency | Binary variable: <3 times per week and ≥3 times per week. |
| Diet | Binary variable measured by healthy diet score. It was measured using 3 food items: 1) total fruit and vegetable intake: >4.5 pieces or servings per day; 2) total fish intake: >2 times per week; 3) processed and red meat intake: ≤2 times of processed meat per week and ≤ 5times of red meat per week. If ≥2 healthy food items were fulfilled, healthy diet score was 1 (more advisable), otherwise healthy diet score was 0 (less advisable). |
| Sleep duration | Continuous variable (hours per day). |
| Weight loss | Binary variable: lose weight and not. |
| None or light activity | Self-reported: UK Biobank physical activity questionnaire. Physical activity levels were classified to: none (no physical activity in the last 4 weeks), low (light DIY activity (e.g., pruning, watering the lawn) only in the past 4 weeks), medium (heavy DIY activity (e.g., weeding, lawn mowing, carpentry and digging), walking for pleasure, or other exercises in the past 4 weeks), and high (strenuous sports in the past 4 weeks). Binary variable: None or light activity with a frequency of once per week or less: 1; Medium or heavy activity, or light activity more than once per week: 0. |
| Slow pace | Binary variable: slow pace and not. |
| Low grip score | Measured using a Jamar J00105 hydraulic hand dynamometer. The maximum of right and left values of grip strength was used. Binary variable: Compared with sex- and body-mass index adjusted cutoffs by Fried et al. |
| Exhaustion | Binary variable: More than half the days or nearly every day and not. |
| BMI | Body weight (kg) divided by the square of height (m^2^). Continuous variable (kg/m^2^). |
| Hba1c | Measured by HPLC analysis on a Bio-Rad VARIANT II Turbo. Continuous variable (mmol/mol). |
| Fall history | Binary variable: had fall history in the last year at baseline and not. |

CVD: Cardiovascular disease; DBP: Diastolic blood pressure; GED: General Educational Development; HbA1c: haemoglobin A1c; HPLC: High-pressure liquid chromatography; HUI3: Health Utilities Index Mark3; MNSI: Michigan Neuropathy Screening Instrument; SBP: Systolic blood pressure.

Table S3 - Baseline characteristics of participants in the ACCORD and UK Biobank after propensity matching

| Variable | Total sample | Non-VNS | VNS | *P* |
| --- | --- | --- | --- | --- |
| ACCORD | | | | |
| Participants | 5984 | 2992 (50.0) | 2992 (50.0) | - |
| Male, No. (%) | 3555 (59.4) | 1790 (59.8) | 1765 (59.0) | 0.528 |
| Age, mean (SD), year | 63.09 (6.54) | 63.14 (6.60) | 63.04 (6.47) | 0.529 |
| White, No. (%) | 4257 (71.1) | 2132 (71.3) | 2125 (71.0) | 0.993 |
| Education, No. (%) |  |  |  | 0.501 |
| Less than high school graduate | 628 (10.5) | 330 (11.0) | 298 (10.0) |  |
| High school grad (or GED) | 1484 (24.8) | 735 (24.6) | 749 (25.0) |  |
| Some college or technical school | 2104 (35.2) | 1036 (34.6) | 1068 (35.7) |  |
| College graduate or more | 1768 (29.5) | 891 (29.8) | 877 (29.3) |  |
| Current smoker, No. (%) | 566 (9.5) | 279 (9.3) | 287 (9.6) | 0.757 |
| Alcohol consumption >2 times/week, No. (%) | 651 (10.9) | 328 (11.0) | 323 (10.8) | 0.868 |
| MNSI score, median [IQR] | 2.00 [1.00, 3.50] | 2.00 [1.00, 4.00] | 2.00 [1.00, 3.00] | 0.525 |
| HUI3 score, median [IQR] | 0.79 [0.59, 0.92] | 0.79 [0.61, 0.92] | 0.79 [0.59, 0.92] | 0.794 |
| BMI, mean (SD), kg/m^2^ | 32.51 (5.32) | 32.53 (5.29) | 32.49 (5.35) | 0.795 |
| HbA1c, mean (SD), mmol/mol ^a^ | 65.94 (10.64) | 66.00 (10.85) | 65.88 (10.44) | 0.655 |
| SBP, mean (SD), mmHg | 135.15 (16.90) | 135.17 (16.82) | 135.12 (16.99) | 0.904 |
| DBP, mean (SD), mmHg | 74.10 (10.42) | 74.05 (10.43) | 74.15 (10.41) | 0.713 |
| Duration of DM, mean (SD), year | 10.76 (7.47) | 10.79 (7.63) | 10.73 (7.32) | 0.735 |
| History of diseases, No. (%) |  |  |  |  |
| CVD | 2037 (34.0) | 1036 (34.6) | 1001 (33.5) | 0.354 |
| Heart failure | 275 (4.6) | 143 (4.8) | 132 (4.4) | 0.537 |
| Foot ulcer | 229 (3.8) | 117 (3.9) | 112 (3.7) | 0.788 |
| Intervention arms, No. (%) |  |  |  | 0.457 |
| Standard glycemia/intensive BP | 699 (11.7) | 360 (12.0) | 339 (11.3) |  |
| Standard glycemia/standard BP | 689 (11.5) | 325 (10.9) | 364 (12.2) |  |
| Intensive glycemia/intensive BP | 688 (11.5) | 341 (11.4) | 347 (11.6) |  |
| Intensive glycemia/standard BP | 710 (11.9) | 369 (12.3) | 341 (11.4) |  |
| Standard glycemia/lipid fibrate | 823 (13.8) | 420 (14.0) | 403 (13.5) |  |
| Standard glycemia/lipid placebo | 799 (13.4) | 382 (12.8) | 417 (13.9) |  |
| Intensive glycemia/lipid fibrate | 794 (13.3) | 409 (13.7) | 385 (12.9) |  |
| Intensive glycemia/lipid placebo | 782 (13.1) | 386 (12.9) | 396 (13.2) |  |
| UK Biobank | | | | |
| Participants | 18222 | 9111 | 9111 |  |
| Male, No. (%) | 11004 (60.4) | 5505 (60.4) | 5499 (60.4) | 0.940 |
| Age, median [IQR], year | 61.9 [56.1, 66.0] | 61.9 [55.9, 66.0] | 61.9 [56.2, 65.9] | 0.831 |
| White, No. (%) | 16347 (89.7) | 8174 (89.7) | 8173 (89.7) | 1.000 |
| TDI, median [IQR] | -1.5 [-3.3, 1.7] | -1.5 [-3.3, 1.7] | -1.5 [-3.3, 1.6] | 0.824 |
| Current smoker, No. (%) | 1764 (9.7) | 880 (9.7) | 884 (9.7) | 0.940 |
| Alcohol consumption >2 times/week, No. (%) | 5846 (32.1) | 2939 (32.3) | 2907 (31.9) | 0.623 |
| Healthy diet, No. (%) | 10416 (57.2) | 5217 (57.3) | 5199 (57.1) | 0.799 |
| Sleep duration, mean (SD), hour/day | 7.0 [6.0, 8.0] | 7.0 [6.0, 8.0] | 7.0 [6.0, 8.0] | 0.979 |
| Weight loss, No. (%) | 5045 (27.7) | 2515 (27.6) | 2530 (27.8) | 0.817 |
| None or light activity, No. (%) | 2915 (16.0) | 1458 (16.0) | 1457 (16.0) | 1.000 |
| Slow pace, No. (%) | 4023 (22.1) | 2013 (22.1) | 2010 (22.1) | 0.972 |
| Low grip score, No. (%) | 6549 (35.9) | 3268 (35.9) | 3281 (36.0) | 0.853 |
| Exhaustion, No. (%) | 3627 (19.9) | 1814 (19.9) | 1813 (19.9) | 1.000 |
| BMI, median [IQR], kg/m^2^ | 30.3 [27.1, 34.3] | 30.4 [27.1, 34.3] | 30.2 [27.0, 34.3] | 0.162 |
| HbA1c, mean (SD), mmol/mmol | 49.4 [42.3, 58.3] | 49.4 [42.5, 58.3] | 49.4 [42.2, 58.2] | 0.290 |
| Fall history, No. (%) | 4571 (25.1) | 2306 (25.3) | 2265 (24.9) | 0.494 |
| History of diseases, No. (%) |  |  |  |  |
| CHD | 3391 (18.6) | 1680 (18.4) | 1711 (18.8) | 0.568 |
| Hypertension | 12872 (70.6) | 6445 (70.7) | 6427 (70.5) | 0.782 |
| Stroke | 971 (5.3) | 489 (5.4) | 482 (5.3) | 0.843 |
| Diabetic complications | 1197 (6.6) | 603 (6.6) | 594 (6.5) | 0.811 |
| Osteoporosis | 100 (0.5) | 52 (0.6) | 48 (0.5) | 0.764 |

Data were presented as mean (SD), median [IQR], or n (%) for continuous and categorical variables, respectively.

^a^ Only HbA1c values expressed in percentage were available in ACCORD trial, and the values were transformed to mmol/mol in correspondence with the units used in UK Biobank.

BMI: Body mass index; CHD: Coronary heart disease; CVD: Cardiovascular disease; DBP: Diastolic blood pressure; GED: General Educational Development; HbA1c: haemoglobin A1c; HUI3: Health Utilities Index Mark3; MNSI: Michigan Neuropathy Screening Instrument; SBP: Systolic blood pressure; VNS: Vitamin and/or nutritional supplements.

Table S4 – Associations of overall VNS and specific VNS with the risk of fall after propensity matching in UK Biobank.

| Main variable | Model 1 ^a^ | | Model 2 ^b^ | | Model 3 ^c^ | |
| --- | --- | --- | --- | --- | --- | --- |
|  | HR (95% CI) | *P* ^d^ | HR (95% CI) | *P* ^d^ | HR (95% CI) | *P* ^d^ |
| Overall VNS | 1.01 (0.92, 1.11) | 0.850 | 1.02 (0.93, 1.11) | 0.814 | 1.02 (0.93, 1.12) | 0.838 |
| Vitamin | 1.09 (0.99, 1.21) | 0.148 | 1.10 (1.00, 1.21) | 0.148 | 1.09 (0.99, 1.20) | 0.223 |
| Vitamin A | 1.06 (0.79, 1.44) | 0.796 | 1.05 (0.77, 1.42) | 0.814 | 1.02 (0.75, 1.39) | 0.960 |
| Vitamin B | **1.38 (1.15, 1.66)** | **0.003** | **1.38 (1.15, 1.65)** | **0.004** | **1.35 (1.13, 1.62)** | **0.009** |
| Vitamin C | 1.02 (0.86, 1.20) | 0.850 | 1.06 (0.90, 1.25) | 0.695 | 1.04 (0.88, 1.23) | 0.838 |
| Vitamin D | 1.20 (0.96, 1.51) | 0.183 | 1.21 (0.96, 1.52) | 0.214 | 1.20 (0.95, 1.52) | 0.257 |
| Vitamin E | 1.12 (0.87, 1.45) | 0.464 | 1.18 (0.91, 1.53) | 0.367 | 1.18 (0.91, 1.52) | 0.394 |
| Folate | **1.31 (1.03, 1.66)** | 0.084 | 1.15 (0.90, 1.46) | 0.405 | 1.13 (0.88, 1.43) | 0.510 |
| Mineral supplements | 0.92 (0.84, 1.01) | 0.152 | 0.94 (0.86, 1.03) | 0.367 | 0.95 (0.87, 1.04) | 0.450 |
| Fish oil | 0.86 (0.78, 0.95) | 0.013 | 0.91 (0.82, 1.00) | 0.148 | 0.92 (0.83, 1.01) | 0.223 |
| Glucosamine | 0.91 (0.80, 1.04) | 0.225 | 0.96 (0.85, 1.09) | 0.700 | 0.99 (0.87, 1.12) | 0.960 |
| Calcium | **1.45 (1.22, 1.72)** | **<0.001** | **1.39 (1.17, 1.66)** | **0.003** | **1.36 (1.14, 1.62)** | **0.008** |
| Iron | **1.51 (1.23, 1.84)** | **<0.001** | **1.31 (1.07, 1.61)** | **0.045** | 1.25 (1.02, 1.54) | 0.116 |
| Zine | 0.95 (0.75, 1.21) | 0.796 | 1.00 (0.78, 1.27) | 0.990 | 0.99 (0.78, 1.27) | 0.960 |
| Selenium | 1.30 (0.98, 1.71) | 0.148 | 1.36 (1.03, 1.80) | 0.116 | 1.39 (1.05, 1.84) | 0.105 |

^a^ Model 1: adjusted for age, gender, and race.

^b^ Model 2: adjusted for age, gender, race, Townsend deprivation index, smoking statue, alcohol consumption, diet, sleep duration, activity, weight loss, grip strength score, walk speed, and exhaustion.

^c^ Model 3: adjusted for variables in model 2 plus BMI, HbA1c, history of CHD, hypertension, stroke, recent fall, and diabetic complication.

^d^ *P*: *P* value was adjusted for multiple comparisons using Benjamini&Hochberg method. BMI: Body mass index; CHD: Coronary heart disease; HbA1c: haemoglobin A1c; VNS: Vitamin and/or nutritional supplements.

Table S5 – Sensitivity analyses based on UK Biobank

| Variable | Sensitivity analysis 1 | | Sensitivity analysis 2 | | Sensitivity analysis 3 | | Sensitivity analysis 4 | | Sensitivity analysis 5 | |
| --- | --- | --- | --- | --- | --- | --- | --- | --- | --- | --- |
|  | HR (95% CI) | *P* ^a^ | HR (95% CI) | *P* ^a^ | HR (95% CI) | *P* ^a^ | HR (95% CI) | *P* ^a^ | HR (95% CI) | *P* ^a^ |
| VNS | 1.03 (0.94, 1.13) | 0.546 | 1.03 (0.94, 1.11) | 0.646 | 1.04 (0.97, 1.12) | 0.409 | 1.02 (0.93, 1.11) | 0.770 | 1.03 (0.95, 1.13) | 0.613 |
| Vitamin | 1.11 (1.01, 1.23) | 0.098 | 1.10 (1.00, 1.20) | 0.103 | **1.10 (1.02, 1.19)** | **0.043** | 1.11 (1.01, 1.23) | 0.102 | 1.11 (1.01, 1.22) | 0.068 |
| Vitamin A | 1.25 (0.94, 1.68) | 0.279 | 1.13 (0.86, 1.48) | 0.532 | 1.17 (0.92, 1.48) | 0.300 | 1.15 (0.86, 1.53) | 0.477 | 1.13 (0.85, 1.49) | 0.585 |
| Vitamin B | **1.32 (1.08, 1.60)** | **0.038** | **1.35 (1.14, 1.60)** | **0.005** | **1.33 (1.15, 1.55)** | **0.001** | **1.36 (1.13, 1.64)** | **0.008** | **1.37 (1.15, 1.64)** | **0.003** |
| Vitamin C | 1.02 (0.86, 1.22) | 0.800 | 1.01 (0.86, 1.18) | 0.950 | 1.04 (0.91, 1.19) | 0.658 | 1.03 (0.88, 1.22) | 0.770 | 1.02 (0.87, 1.19) | 0.912 |
| Vitamin D | **1.35 (1.08, 1.68)** | **0.038** | 1.26 (1.03, 1.55) | 0.078 | **1.31 (1.10, 1.57)** | **0.010** | 1.26 (1.01, 1.56) | 0.103 | 1.28 (1.04, 1.58) | 0.066 |
| Vitamin E | 1.13 (0.87, 1.48) | 0.475 | 1.15 (0.91, 1.45) | 0.433 | 1.20 (0.97, 1.48) | 0.152 | 1.15 (0.90, 1.47) | 0.405 | 1.21 (0.95, 1.53) | 0.225 |
| Folate | 1.18 (0.91, 1.52) | 0.394 | 1.27 (1.02, 1.57) | 0.078 | **1.27 (1.06, 1.54)** | **0.028** | 1.31 (1.05, 1.64) | 0.085 | 1.23 (0.99, 1.53) | 0.135 |
| Mineral supplement | 0.96 (0.87, 1.05) | 0.475 | 0.96 (0.88, 1.04) | 0.465 | 0.97 (0.90, 1.05) | 0.513 | 0.94 (0.86, 1.03) | 0.267 | 0.97 (0.89, 1.06) | 0.613 |
| Fish oil | 0.95 (0.86, 1.05) | 0.475 | 0.92 (0.84, 1.01) | 0.174 | 0.93 (0.85, 1.01) | 0.137 | 0.92 (0.84, 1.02) | 0.188 | 0.94 (0.85, 1.03) | 0.283 |
| Glucosamine | 0.94 (0.82, 1.08) | 0.475 | 0.96 (0.85, 1.08) | 0.588 | 1.01 (0.90, 1.12) | 0.930 | 0.95 (0.84, 1.08) | 0.525 | 0.98 (0.87, 1.11) | 0.888 |
| Calcium | 1.24 (1.03, 1.49) | 0.075 | **1.38 (1.18, 1.62)** | **0.001** | **1.36 (1.18, 1.57)** | **<0.001** | **1.39 (1.18, 1.65)** | **0.001** | **1.41 (1.20, 1.66)** | **<0.001** |
| Iron | **1.33 (1.07, 1.64)** | **0.038** | **1.35 (1.12, 1.62)** | **0.008** | **1.31 (1.11, 1.54)** | **0.006** | 1.21 (0.98, 1.50) | 0.176 | **1.33 (1.10, 1.61)** | **0.014** |
| Zine | 0.90 (0,70, 1.18) | 0.519 | 0.95 (0.76, 1.20) | 0.739 | 0.96 (0.78, 1.18) | 0.739 | 0.97 (0.76, 1.23) | 0.770 | 1.00 (0.79, 1.26) | 0.980 |
| Selenium | **1.47 (1.12, 1.94)** | **0.038** | 1.34 (1.03, 1.74) | 0.078 | **1.36 (1.07, 1.73)** | **0.028** | 1.35 (1.02, 1.79) | 0.102 | **1.43 (1.09, 1.86)** | **0.033** |

Sensitivity analysis 1: excluding 2,167 patients who experienced ≥2 fall in last year (n=19,322).

Sensitivity analysis 2: adjusted for frailty phenotype instead of weight loss, exhaustion, walking speed, physical activity, response, and grip strength score.

Sensitivity analysis 3: filling in missing value of other covariables with multiple imputation.

Sensitivity analysis 4: excluding 1,399 patients with diabetic complications at baseline (n=20,090).

Sensitivity analysis 5: excluding 89 patients who experienced inpatient fall during the first year of follow-up (n=21,400).

^a^ *P*: *P* value was adjusted for multiple comparisons using Benjamini&Hochberg method.

VNS: Vitamin and/or nutritional supplements.

# Data Availability Statement

The datasets analyzed for this study can be found in the National Heart, Lung, and Blood Institute Biologic Specimen and Data Repository (https://biolincc.nhlbi.nih.gov/studies/accord/ for ACCORD trial) and UK Biobank (https://www.ukbiobank.ac.uk/) but restrictions apply to the availability of these data, which were used under license for the current study, and so are not publicly available. Data are however available from the authors upon reasonable request and with permission of the National Heart, Lung, and Blood Institute Biologic Specimen and Data Repository and UK Biobank.
